# Amoebae, Giant Viruses, and Virophages Make Up a Complex, Multilayered Threesome

**DOI:** 10.3389/fcimb.2017.00527

**Published:** 2018-01-11

**Authors:** Jan Diesend, Janis Kruse, Monica Hagedorn, Christian Hammann

**Affiliations:** Ribogenetics Biochemistry Lab, Department of Life Sciences and Chemistry, Jacobs University Bremen, Bremen, Germany

**Keywords:** *Acanthamoeba polyphaga* mimivirus (APMV), virophage, nucleocytoplasmatic large DNA virus (NCLDV), mimivirus, pathogen defense

## Abstract

Viral infection had not been observed for amoebae, until the *Acanthamoeba polyphaga* mimivirus (APMV) was discovered in 2003. APMV belongs to the nucleocytoplasmatic large DNA virus (NCLDV) family and infects not only *A. polyphaga*, but also other professional phagocytes. Here, we review the *Megavirales* to give an overview of the current members of the *Mimi*- and *Marseilleviridae* families and their structural features during amoebal infection. We summarize the different steps of their infection cycle in *A. polyphaga and Acanthamoeba castellani*. Furthermore, we dive into the emerging field of virophages, which parasitize upon viral factories of the *Megavirales* family. The discovery of virophages in 2008 and research in recent years revealed an increasingly complex network of interactions between cell, giant virus, and virophage. Virophages seem to be highly abundant in the environment and occupy the same niches as the *Mimiviridae* and their hosts. Establishment of metagenomic and co-culture approaches rapidly increased the number of detected virophages over the recent years. Genetic interaction of cell and virophage might constitute a potent defense machinery against giant viruses and seems to be important for survival of the infected cell during mimivirus infections. Nonetheless, the molecular events during co-infection and the interactions of cell, giant virus, and virophage have not been elucidated, yet. However, the genetic interactions of these three, suggest an intricate, multilayered network during amoebal (co-)infections. Understanding these interactions could elucidate molecular events essential for proper viral factory activity and could implicate new ways of treating viruses that form viral factories.

## Introduction to giant viruses

**Figure 1:**
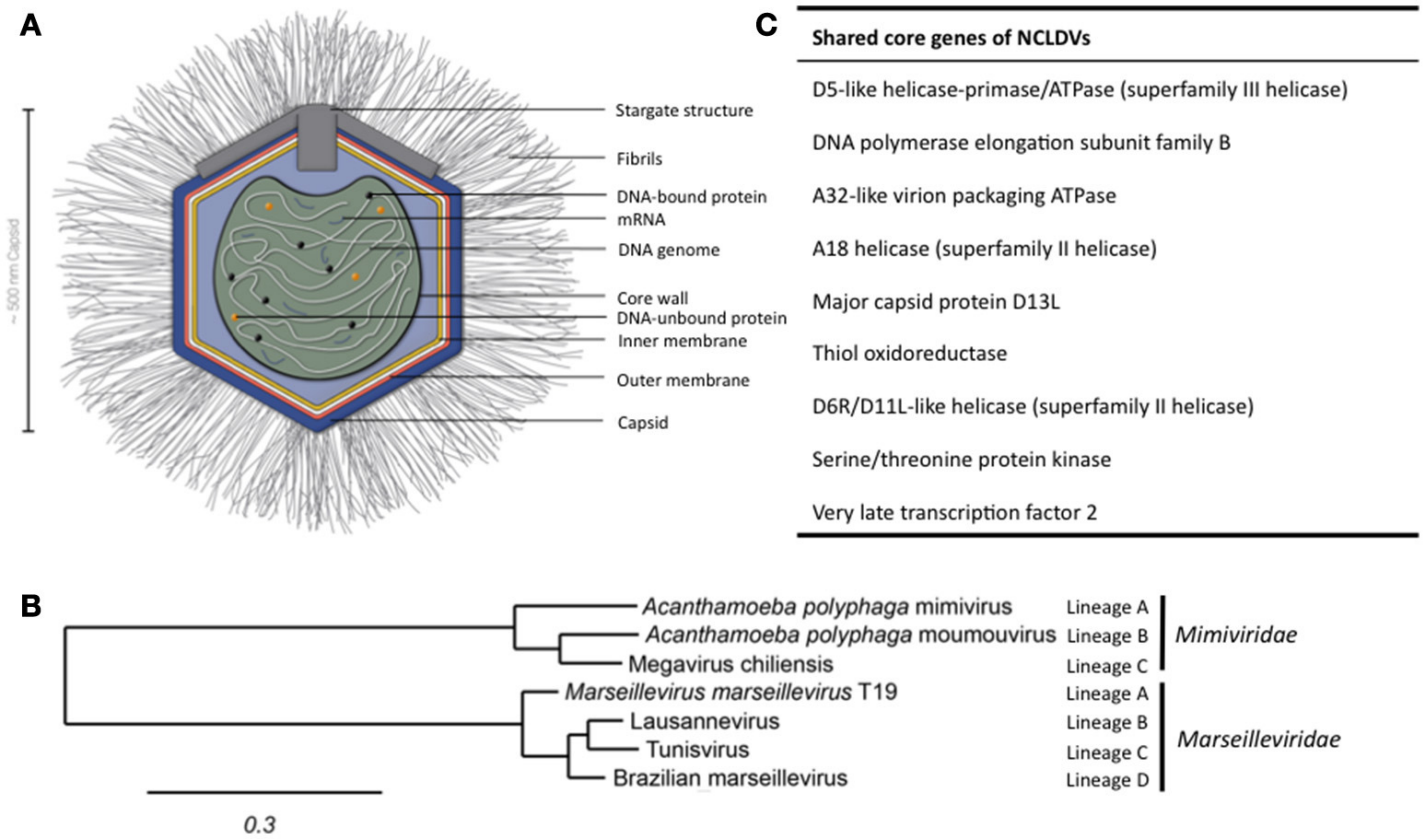
Structure of APMV and the core genes and relationship of giant viruses. **(A)** Viral particles of APMV feature a viral core with the genome, mRNAs, and prefabricated proteins. This core is surrounded by the indicated membranes and the capsid structure that contains a pentagonal, star-shaped structure termed “stargate,” which is involved in the release of the viral core into the host cell's cytosol upon phagocytosis. The capsid is decorated with a compact layer of fibrils. For details see main text. **(B)** Cladogram displaying the relationships of the different lineages of the *Mimiviridae* and *Marseilleviridae*. Since the discovery of APMV, over 100 new mimivirus strains have been characterized using samples of various origins in amoebal co-culture methods (Pagnier et al., 2013; Khalil et al., 2016a,b). All *Mimiviridae* share a capsid size between 370 and 600 nm and a 1.02–1.26 Mb AT-rich genome which encodes about 1.000 putative proteins (Colson et al., 2017). Based on sequence homology, the *Mimiviridae* can be divided into three distinct lineages: lineage A with APMV as prototype and a total of 18 members, as reviewed recently (Colson et al., 2017), lineage B with the moumouvirus as prototype and four additional members (Yoosuf et al., 2012; Colson et al., 2017), and lineage C with Megavirus chiliensis as prototype and a total of 12 members (Arslan et al., 2011; Colson et al., 2017). The tree was created using the sequences of the D13 major capsid proteins of the indicated prototype viruses using Phylogeny.fr, with the relative evolutionary distance indicated (Dereeper et al., 2008, 2010). **(C)** List of nine genes conserved throughout all NCLDV families.

The discovery of giant viruses in the early 2000s led to a mind shift in the field of virology with respect to the potential origins of viruses (La Scola et al., 2003; Raoult et al., 2004). Originally, viruses were thought of as submicroscopic particles with a self-evident denial that viruses might exist, whose size would be large enough to be resolved with a simple light microscope (Lwoff, 1957; Raoult, 2013). Due to this mindset, the large, gram-positive particles in an *Acanthamoeba polyphaga* population were at first erroneously classified as bacteria (Birtles et al., 1997; La Scola et al., 2003; Raoult et al., 2007). Only the absence of ribosomal DNA in the presumed bacterium, led to the discovery and definition of the *A. polyphaga* mimivirus (APMV) in 2003 (La Scola et al., 2003). The acronym mimivirus (for mimicking microbe) reflects the resemblance to bacteria upon gram staining. At the same time, the discovery of APMV was the first ever report of a virus infecting amoebae. Amongst other features that are detailed below, APMV is unusual as it contains a large genome of 1.14 Mbp, thereby even surpassing the genome size of some bacterial species (Raoult et al., 2004). APMV particles are characterized by an up to 700 nm large capsid (Figure 1A), which is well above the resolution of a simple light microscope. Once it was established that giant DNA viruses of amoebae exist, many more such viruses, belonging to the nucleoplasmatic large DNA viruses (NCLDV) were found in the environment, as well as within a wide range of host organisms from humans, monkeys, and oysters (Boughalmi et al., 2013a; Dornas et al., 2014; Andrade et al., 2015). *Ex vivo* studies of human cell lines revealed that APMV is capable of infecting myeloid and mononuclear blood cells and interferes with the type I Interferon system (Silva et al., 2014). In addition, a distantly APMV-related NCLDV family member has been shown to productively infect T-lymphocytes under laboratory conditions (Popgeorgiev et al., 2013). In 2008, a small particle called Sputnik 1 (La Scola et al., 2008) was discovered in *A. polyphaga*, which parasitizes viral factories of giant viruses. Due to the functional similarity to bacteriophages in mediating lateral gene transfer, Sputnik was classified as a virophage (La Scola et al., 2008). Here, we will review the expanding family of virophages and discuss the implications for giant virus reproduction inside amoebae.

## The diverse families of giant viruses that infect amoebae

The discovery of APMV sparked the interest in giant viruses and spawned a contemporary research field of its own (La Scola et al., 2003). Up until today, two giant virus families belonging to the NCLDV have been described that primarily infect amoebae: the *Mimiviridae* and the *Marseilleviridae* (Figure 1B). The latter has the *A. polyphaga* marseillevirus (APMaV) as founding member, which was discovered in 2009 (Boyer et al., 2009; Colson et al., 2013). In the last decade, nine additional viruses have been associated with the *Marseilleviridae* group (Colson et al., 2017). The *Acanthamoeba castellani* lausannevirus (ACLaV) was discovered by incubating water from the Seine river in France with *A. castellani*, a close relative of *A. polyphaga* (Thomas et al., 2011). ACLaV is the first known giant virus to encode histone-like proteins, which could point towards a DNA packaging mechanism similar to eukaryotes (Thomas et al., 2011). The Cannes 8 virus (Ca8V) (La Scola et al., 2010) and the Senegal virus (SNGV) (Lagier et al., 2012) have been isolated using similar co-culture methods and are grouped with the *Marseilleviridae*. The icosahedral capsid of the *Marseilleviridae* is between 190 and 250 nm in diameter (Colson et al., 2013). Like the genome of the *Mimiviridae*, the 370,000 bp dsDNA genome is encased in a lipid bilayer and encodes about 450 proteins (Boyer et al., 2009; La Scola et al., 2010; Thomas et al., 2011; Lagier et al., 2012). Both, *Mimiviridae* and *Marseilleviridae*, share only nine core genes with all NCLDVs (Figure 1C) and 180 genes are shared with at least two of the NCLDV families (Yutin et al., 2009; Yutin and Koonin, 2012). Based on the discovery of APMV and its complex genome, it was suggested to incorporate viruses into the tree of life by defining them as capsid-encoding organisms contrary to the ribosome-encoding organisms, which are represented by eukarya, bacteria, and archaea (Raoult and Forterre, 2008).

## APMV—the best studied giant virus of amoebae

APMV was the first giant virus to be discovered (La Scola et al., 2008) and confronted the scientific community with features never observed in a virus before. Its capsid size and genetic complexity with many genes usually found in eukaryotic and prokaryotic cells challenged the Lwoff's characteristics of a virus (Raoult et al., 2004; Raoult and Forterre, 2008). The AT-rich 1.14 Mbp APMV genome features an impressive number of 979 protein-encoding genes in a dense arrangement (Raoult et al., 2004; Legendre et al., 2011). Several of its genes are only found in giant viruses of amoebae and code for virus-atypical proteins involved in DNA repair, protein folding, tRNA synthesis and translation, and more (Raoult et al., 2004). In addition, the APMV genome displays some plasticity and encodes self-splicing introns, inteins, and a specific set of mobile genetic elements called transpovirons (Desnues et al., 2012). Furthermore, the genome contains many genes likely acquired via horizontal gene transfer, paralogous genes, and so called ORFans, genes that encode proteins with unknown function (Suhre, 2005; Filée et al., 2007; Moreira and Brochier-Armanet, 2008; Forterre, 2010). Many of these genes are shared with the poxviruses, phycodnaviruses, and other NCLDVs (Filée et al., 2007). ORFans represent roughly 50% of genes and about 40% of the APMV proteome, which results in a high number of factors with unknown functions that might act during viral replication and morphogenesis (Renesto et al., 2006). Alike “classical” viruses, APMV genes are partly under the control of early and late stage-specific promoters (Raoult et al., 2004; Suhre et al., 2005).

The APMV particles possess remarkable structural features, separating them from the classical structures of viruses (Figure 1A). In its center, the viral DNA, mRNAs and proteins are packed into the core compartment (Xiao et al., 2009; Kuznetsov et al., 2013) and enclosed by a lipid membrane. Among the pre-packed proteins are 12 enzymes involved in transcription, five in DNA repair, two in RNA modification, and five in protein modification (Renesto et al., 2006). The central compartment is surrounded by an approximately 340 nm-large lipid bilayer and a secondary bilayer directly underneath an icosahedral capsid. This is comprised of major capsid proteins and features a five-branch proteinaceous structure, the “stargate,” at one vortex (Kuznetsov et al., 2013). The capsid itself is covered by a compact layer of about 120–140 nm long, heavily glycosylated fibrils, which potentially facilitate the attachment of APMV to its host cells (Rodrigues et al., 2015).

As of now, only four fiber associated proteins (FAP1-4) have been functionally associated with either fibril biosynthesis or as components of the fibrils (Sobhy et al., 2015). FAP1 is an aryl alcohol oxidase, which catalyzes the degradation of lignin or lignin derivatives. This suggests that APMV might also be able to infect lignin-containing algae (Klose et al., 2015; Rodrigues et al., 2015). However, the fibrils and associated proteins are not essential for the productive infection of amoebae: during long-term intraamoebal culture (150 generations), the responsible genes are lost (Boyer et al., 2011; Rodrigues et al., 2015). This indicates that the genomic complexity of APMV might be maintained to allow for a broad host range. If so, only a subset of its diverse molecular tools would come in use to enter and infect individual hosts.

## Infection cycles of giant viruses in amoebae

Even though the replication cycle of most giant viruses differ in aspects like nuclear involvement, duration, assembly, and release of the viral progeny, key steps in the infection appear to be conserved, as summarized recently (Colson et al., 2017). For example, all known giant viruses enter the host cell by phagocytosis and release their DNA into the cytosol in a similar manner (Ghigo et al., 2008). Furthermore, viral replication takes place in specialized endoplasmatic reticulum (ER)-derived compartments that are found in the cytosol and are called viral factories (Xiao et al., 2009; Mutsafi et al., 2010; Kuznetsov et al., 2013).

After uptake, the virus resides in a *de-novo* phagosome. Subsequently, the phagosomal and viral membranes fuse, which allows the release of the viral core, that contains the genome, proteins, and mRNAs into the cytosol (Zauberman et al., 2008; Mutsafi et al., 2010). Alike the well-described poxvirus (Broyles, 2003), the structural integrity of the viral core seems to be retained until viral factories arise (Claverie et al., 2009; Mutsafi et al., 2010). Intriguingly, recent experiments suggest that viral transcription might be initiated already before the release of the viral core (Mutsafi et al., 2014). Once in the cytosol, replication of the viral genome begins immediately and the expression of early stage genes leads to the formation of early viral factories (Suzan-Monti et al., 2007; Mutsafi et al., 2013, 2014). The replication cycle is confined to the cytosol, again a trait shared with the poxvirus (La Scola et al., 2003; Claverie et al., 2009). This also suggests that giant viruses (like the poxvirus) must carry transcription complexes to initiate transcription immediately after infection (Resch et al., 2007; Claverie et al., 2009). In later stages of infection, these viral factories merge into one large cytosolic compartment for replication and capsid assembly (Suzan-Monti et al., 2007; Mutsafi et al., 2014). It should be noted that viral factories are not chaotic, but rather appear to feature distinct assembly lines for their progeny. The viral factory is made up of functional regions playing discrete roles in replication, capsid assembly, DNA packaging, and attachment of fibrils (Suzan-Monti et al., 2007; Mutsafi et al., 2014). In the outermost layer of the viral factory, the internal membrane layers of APMV are assembled from host-derived membrane vesicles, which are thought to rupture, thereby forming open single-layer membrane sheets (Mutsafi et al., 2013). Capsid assembly occurs around these membrane sheets and is scaffolded by the major capsid protein L425 (Mutsafi et al., 2013). Upon capsid formation, the genome is deposited into the empty viral particle through a transient interstice distal from the “stargate” structure (Zauberman et al., 2008). There is little evidence for a nuclear stage of giant viruses. However, the nuclei of *A. polyphaga* and *A. castellani* exhibit transient changes in their morphology during the early stages of infection with members of the *Marseilleviridae* family (Arantes et al., 2016). This indicates that nuclear host factors might play a role in the APMV replication, a notion that is supported by a two-fold decrease of the nuclear size in infected *A. polyphaga* cells (Colson et al., 2017). This might be due to a substantial redistribution of nuclear factors for viral replication, transcription or other processes (Colson et al., 2017). Albeit indirectly, this scenario is supported by data on the cytoplasmic replication of the Vaccinia virus (a poxvirus), to which mimivirus replication bears similarities (Mutsafi et al., 2010) and for which the involvement of nuclear enzymes has been demonstrated (Oh and Broyles, 2005).

## Virophages as parasites of the megavirales

The description of *Megavirales* infection of amoebae was followed by the discovery of the fascinating virophage Sputnik in 2008 (La Scola et al., 2008). Sputnik was found infecting the viral factories of the mamavirus, a close relative of APMV (La Scola et al., 2008). Replication of the Sputnik virophages inside APMV-infected *A. castellani* cells is deleterious to APMV replication and results in abortive DNA replication and disruption of capsid biogenesis (La Scola et al., 2008). There is an ongoing discussion on the classification of virophages, that are denoted in several articles as satellite viruses (Krupovic and Cvirkaite-Krupovic, 2011; Blanc et al., 2015; Koonin and Krupovic, 2017). Satellite viruses are characterized by their dependency on factors of a helper virus. However, the Sputnik genomes itself encodes factors involved in viral replication (La Scola et al., 2008), suggesting that Sputnik can be classified as a virus, rather than a defective viral particle or sub-viral agent (Fischer, 2011; Desnues and Raoult, 2012).

All known members of the virophage family parasitizing on giant viruses are categorized into the large virus-dependent or -associated (*Lavida*-)*viridae* family that is divided into the *Sputnikvirus* and *Mavirus* genera (Krupovic et al., 2016). At the species level, the *Sputnikvirus* genus can be differentiated into the APMV-dependent Sputnik virophage and the APMV-dependent Zamilon virophage (Table 1), while *Mavirus* genus contains only the *Cafeteria roenbergensis* virus (CroV)-dependent mavirus (Krupovic et al., 2016).

**Table 1:** Overview of the *Mimiviridae* and *Marseilleviridae* families and experimentally shown virophage infections.

**Family**	**L^a^**	**Name**	**Place discovered**	**Size [nm]**	**References**	**GenBank Acc. No**.	**Genome size [kbp]**	**No. of ORFs (predicted)**	**GC%**	**Virophage**	**References**
*Mimiviridae*	A	*A. polyphaga* mimivirus	Bradford, England	750	La Scola et al., 2003	NC_014649.1	1,182	979	28	Sputnik	La Scola et al., 2008
	A	*A. polyphaga* mamavirus	Paris, France	750	Colson et al., 2011	JF801956.1	1,192	1023	28	Sputnik	La Scola et al., 2008
	A	Hirudovirus	Tunisia	520	Boughalmi et al., 2013a	KF493731.1	1,155	992	28	No report	
	A	Niemeyer virus	Belo Horizonte, Brazil	620	Boratto et al., 2015	KT599914.1	1,299	1003	28	No report	
	A	Samba virus	Negro River, Brazil	570	Campos et al., 2014; Assis et al., 2015	KF959826.2	1,181	971	28	Rio Negro	Campos et al., 2014
	A	Amazonian virus	Negro River, Brazil		Assis et al., 2015	KM982403	1,180	979	27	No report	
	A	Kroon virus	Lagoa Santa, Brazil		Assis et al., 2015	KM982402.1	1,222	944	27	No report	
	A	Oyster virus	Florianópolis, Brazil		Assis et al., 2015	KM982401.1	1,200	948	27	No report	
	A	Pointe-Rouge 1 virus	Marseille, France	390	La Scola et al., 2010	LN871174.1	1,151			Sputnik	Gaia et al., 2013
	A	Longchamps virus		450	La Scola et al., 2010	LN871173.1	1,104			Sputnik	Gaia et al., 2013
	A	Fauteuil virus		600	La Scola et al., 2010	LN871163.1	1,181			Sputnik	Gaia et al., 2013
	A	Terra2 virus	Marseille, France	370	La Scola et al., 2010; Yoosuf et al., 2014b	KF527228.1	1,167	890	28	Sputnik	Gaia et al., 2013
	A	Pointe-Rouge 2 virus	Marseille, France	500	La Scola et al., 2010	LN871172.1	1,163			Sputnik	Gaia et al., 2013
	A	Lactour virus		450	La Scola et al., 2010	CXOL00000000.1^*^	1,181			Sputnik	Gaia et al., 2013
	A	Lentille virus	Marseille, France	500	La Scola et al., 2010	AFYC00000000.1^*^	1,193	807		Sputnik	La Scola et al., 2010
	A	Shirakomae virus	Nagano, Japan		Takemura et al., 2016	AP017645.1	1,183	996		No report	
	A	Kasaii virus	Tokyo, Japan		Takemura et al., 2016	AP017644.1	1,183	996		No report	
	A	Bombay virus	Mumbai, India	435	Chatterjee et al., 2016b	KU761889.1	1,182	898	28	No report	
	B	*A. polyphaga* moumouvirus	South-East France	420	La Scola et al., 2010; Yoosuf et al., 2012	NC020104.1	1,021	930		Sputnik	Gaia et al., 2013
	B	Monve virus		390	La Scola et al., 2010	JN885994-6001^*^	1,015			Sputnik	Gaia et al., 2013
	B	Saudi moumouvirus	Jeddah, Saudi Arabia	500	Bajrai et al., 2016	KY110734.1	1,046	868	26	No report	
	B	Goulette virus			Boughalmi et al., 2013c	KC008572.1	1,017	979		No report	
	C	Megavirus chiliensis	Las Cruces, Chile	700	Arslan et al., 2011	JN258408.1	1,259	1120	25	No report	
	C	LBA111 virus	Tunisia	550	Saadi et al., 2013a	JX885207.1	1,231	1183		No report	
	C	Courdo 11 virus	Saint-Raphael, France	450	La Scola et al., 2010; Yoosuf et al., 2014a	JX975216.1	1,246	1166		Sputnik	Gaia et al., 2013
	C	Courdo 7 virus	France	400	La Scola et al., 2010	JN885990-3	1,000			Sputnik	Gaia et al., 2013
	C	Terra1 virus	Marseille, France	420	La Scola et al., 2010; Yoosuf et al., 2014b	KF527229.1	1,234	1055	25	Sputnik	Gaia et al., 2013
	C	Shan virus	Marseille, France	640	Saadi et al., 2013b	LN868520.1	1,259			No report	
	C	Courdo5 virus		400	La Scola et al., 2010	LN868540.1	0,922			Sputnik	Gaia et al., 2013
	C	Powai Lake megavirus	Mumbai, India	425	Chatterjee et al., 2016a	KU877344.1	1,209	996	25	No report	
	C	Bus virus		400	La Scola et al., 2010	LN868539.1	1,229			Sputnik	Gaia et al., 2013
	C	Avenue 9 virus			Boughalmi et al., 2013c	LN867403.1	1,214			No report	
	C	Montpellier 3 virus	Montpellier, France	370	La Scola et al., 2010	LN868518.1	1,243			Sputnik	Gaia et al., 2013
	C	Mont1 virus	Tunisia	500	Boughalmi et al., 2013c					Zamilon	Gaia et al., 2014
*Marseilleiviridae*	A	*Marseillevirus marseillevirus* T19	Paris, France	250	Boyer et al., 2009	NC_013756	0,368	457	45	No report	
	A	Giant blood marseillevirus	Marseille, France	220	Popgeorgiev et al., 2013	PRJNA185405^*^	0,357	617	45	No report	
	A	Cannes8 virus	Cannes, France	190	La Scola et al., 2010; Aherfi et al., 2013	KF261120	0,374	484		No report	
	A	Senegalvirus	N'diop, Senegal	200	Lagier et al., 2012	JF909596-602^*^	0,373			No report	
	A	Melbournevirus	Melbourne, Australia	200	Doutre et al., 2014	KM275475.1	0,369	403	45	No report	
	B	Lausannevirus	Seine River, France	200	Thomas et al., 2011	NC_015326	0,347	450		No report	
	B	Port-Miou virus	Port-Miou Calanque, France	200	Doutre et al., 2015	KT428292.1	0,349	410		No report	
	B	Noumeavirus	Noumea, New Caledonia	200	Fabre et al., 2017	NC_033775	0,376	452	43	No report	
	C	Tunisvirus	Tunis, Tunisia	250	Boughalmi et al., 2013c; Aherfi et al., 2014	KF483846.1	0,380	484	43	No report	
	C	Insectomime virus	Tunis, Tunisia	225	Boughalmi et al., 2013b	KF527888.1	0,387	477	43	No report	
	C	Tokyovirus A1	Arakawa River, Japan	200	Takemura, 2016	AP017398.1	0,373	487		No report	
	D	Brazilian marseillevirus	Belo Horizonte, Brazil	250	Dornas et al., 2015, 2016	NC_029692	0,362	491	43	No report	
	E	Golden marseillevirus	Guaíba Lake, Brazil	200	Dos Santos et al., 2016	KT835053.1	0,361	483	43	No report	

* *Genomes with separate available contigs or only raw sequencing data*.

a *Lineage*.

Virophage replication has been extensively studied in particular for Sputnik, Zamilon and mavirus. Studies on amoebae infected with different mimiviruses revealed that Sputnik virophages can parasitize mimiviruses from all *Mimiviridae* lineages but apparently not the *Marseilleviridae* lineages (Gaia et al., 2013). Sputnik replicates inside mamavirus-infected *A. castellani* cells within the viral factories, nonetheless, with different kinetics as the mamavirus and at multiple hot spots inside the factory (La Scola et al., 2008). In APMVs viral factories, Sputnik infection results in the emergence of newly generated particles 6 h post infection with a concomitant decrease of infective APMV particles (Ogata and Claverie, 2008). The 18,343-kilobase circular dsDNA genome of Sputnik possesses 21 partly overlapping open-reading frames (ORFs) encoding for several factors involved in DNA replication (La Scola et al., 2008). Interestingly, four of the ORFs are strongly homologous to APMV-encoded genes (La Scola et al., 2008; Gaia et al., 2013). Since Sputnik virophages encodes a lambda-type integrase, the molecular tools for genomic integration are present (La Scola et al., 2008). Indeed, an integration of the Sputnik genome into the genome of the Lentille virus, a relative of APMV, could be observed experimentally (Desnues et al., 2012). There is no indication of Sputnik genome integration into the host cell genome, in line with the lack of indications for a nuclear phase.

The Zamilon virophage (belonging to the *Sputnikvirus* genus) was discovered together with the Mont1 mimivirus in soil samples from Tunisia (Boughalmi et al., 2013a; Gaia et al., 2014). The 60 nm-wide, spherical virophage carries a 17,276 bp dsDNA genome encoding 20 genes. Although Zamilon shares 76% of its genomic sequence with Sputnik, Zamilon can only infect lineages B and C (Gaia et al., 2014). Furthermore, the tv_L8 protein, encoded in the transpovirons of the Monve mimivirus, shares significant homology with the ORF8-encoded protein of Zamilon (Gaia et al., 2014). This suggests that an exchange of genetic material can in principle occur between the giant virus and the Zamilon virophage within co-infected amoebae, although this has not been observed experimentally so far.

The Maverick-related virus (mavirus), lonely member of the *Mavirus* genus, parasitizes the viral factories of CroV that infects the marine heterotrophic nanoflagellate *C. roenbergensis* (Fischer et al., 2010; Fischer and Suttle, 2011). Although this review is predominantly concerned with infection of amoebae, mavirus is included here for its unique features for a virophage. Its 19,063 bp circular genome possesses 20 ORFs including a retroviral integrase, an unsual, protein-primed DNA polymerase, plus four additional proteins, all of which are also found conserved in Maverick/Polinton (MP) retroelements (Fischer and Suttle, 2011; Krupovic et al., 2014, 2016). Additionally, the termini of the mavirus genome consist of long terminal repeats similar to those found in MP retroelements (Yutin et al., 2013; Krupovic et al., 2016). Both findings suggest that these retroelements might have originated from mavirus genome integration events in mavirus co-infected cells (Fischer and Suttle, 2011; Krupovic et al., 2016). Nonetheless, this hypothesis for the origins of MP retroelements remains to be tested experimentally. Fischer and Hackl (2016) succeeded to monitor the integration of mavirus into the *C. roenbergensis* genome by co-infection with a low multiplicity of infection of CroV. Intriguingly, genes in the mavirus genome possess promoter sequences similar to the late stage promoter of CroV (Fischer and Hackl, 2016). As a consequence, re-infection of *C. roenbergensis* carrying the integrated mavirus genome with CroV resulted in inhibition of CroV DNA replication, concomitantly with an increased survival of *C. roenbergensis* (Fischer and Hackl, 2016).

Other virophages have been discovered by metagenomic analysis of water samples [e.g., the Organic Lake virophage (Yau et al., 2011), the Yellowstone Lake virophages (Zhou et al., 2013, 2015)]. However, the viral and cellular host for these remain to be determined (Krupovic et al., 2016), unlike the situation of the Rio Negro virophage that has the Samba virus as viral host (Campos et al., 2014).

## Outlook

Since the discovery of its first member APMV in 2003, new giant viruses are discovered continuously in samples from all over the world and added to the *Megavirales* family. The addition of virophages as parasites of giant viruses, their high abundance in the environment, and the genetic interactions between cell, giant virus, and virophage, suggest an intricate, multilayered network during amoebal co- and super-infections. Future studies of these dynamic interactions could elucidate the inner mechanics of viral factories.

## Author contributions

All authors designed the mini review. JD: generated the figures and drafted the text; MH and CH: wrote the manuscript.

### Conflict of interest statement

The authors declare that the research was conducted in the absence of any commercial or financial relationships that could be construed as a potential conflict of interest.
